# Protective Effects of Coumestrol on Metabolic Dysfunction and Its Estrogen Receptor-Mediated Action in Ovariectomized Mice

**DOI:** 10.3390/nu15040954

**Published:** 2023-02-14

**Authors:** Song Park, Kyu-Sang Sim, Wan Heo, Jun-Ho Kim

**Affiliations:** 1 Department of Food Science and Biotechnology, Andong National University, Andong 36729, Republic of Korea; 2 Biomaterials Research Institute, Kyochon F&B, Andong 36618, Republic of Korea; 3 Department of Food Science and Engineering, Seowon University, Cheongju 28674, Republic of Korea

**Keywords:** coumestrol, estrogen receptor, metabolic dysfunction, postmenopausal disorders, ovariectomized mouse

## Abstract

Coumestrol, a phytoestrogen compound found in various plants, has been shown to act as a potent estrogen receptor (ER) agonist, with a higher binding affinity for ERβ than for ERα. However, there is currently limited information regarding its beneficial effects in postmenopausal disorders and its ER-mediated mechanisms. Herein, we investigated the effects of coumestrol (subcutaneous or oral treatment) on metabolic dysfunction in ovariectomized (OVX) mice fed a high-fat diet, in comparison with the effects of 17β-estradiol (E2) replacement. Coumestrol was administered daily at a dose of 5 mg/kg for 10 weeks. Coumestrol treatment through the subcutaneous route stimulated uterine growth in OVX mice at a level lower than that of E2. E2 and coumestrol prevented body fat accumulation, adipocyte hypertrophy, and hepatic steatosis, and enhanced voluntary physical activity. Coumestrol showed estrogen-mimetic effects in the regulation of the protein expressions involved in browning of white fat and insulin signaling, including increased hepatic expression of fibroblast growth factor 21. Importantly, the metabolic effects of coumestrol (oral administration at 10 mg/kg for 7 weeks) were mostly abolished following co-treatment with an ERβ-selective antagonist but not with an ERα-selective antagonist, indicating that the metabolic actions of coumestrol in OVX mice are primarily mediated by ERβ. These findings provide important insights into the beneficial effects of coumestrol as a phytoestrogen supplement for the prevention and treatment of postmenopausal symptoms.

## 1. Introduction

Endogenous estrogen deficiency in women after menopause induces, not only impairment of reproductive functions, but also chronic metabolic disorders, including obesity, diabetes, and cardiovascular disease [1]. Phytoestrogens have been widely studied over the past few decades as an effective and safe therapeutic strategy for postmenopausal disorders. Similarly to human estrogens, the primary mode of action of phytoestrogens is initiated by binding to the estrogen receptor (ER), including both subtypes alpha (ERα) and beta (ERβ), and subsequent transcriptional regulation. The majority of in vitro studies have found that phytoestrogens have a higher binding affinity for ERβ than for ERα [2,3,4], suggesting their potential biological effects as ERβ-mediated. Among the various phytoestrogen compounds identified to date, coumestrol, a common phytoestrogen found in various plant sources, such as soybeans, clover, and alfalfa sprouts, is considered one of the most potent ER agonists [5]. In addition to the effects of coumestrol on ERβ activation, our recent comparative study of 15 known phytoestrogens showed coumestrol to have the highest ERα-mediated activity, both in an in vitro ERα transactivation assay and an in vivo uterotrophic assay [6].

With increasing scientific interest in coumestrol as a safer alternative to estrogen replacement therapy, several studies have investigated its protective effects against postmenopausal diseases in ovariectomized (OVX) rodent models. In these studies, coumestrol was shown to display anti-cancer [7], neuroprotective [8], osteoprotective [9], and glycogenolytic [10] activities. Nogowski et al. [10] found that coumestrol enhanced lipid synthesis in the liver and skeletal muscle, while simultaneously stimulating the uterine tissue in OVX rats, suggesting that the effects of coumestrol on lipid metabolism may be independent of its estrogenic action. The same research group also reported that coumestrol did not affect plasma insulin levels but inhibited the binding capacity of insulin receptors as well as the glycogen content in the liver of OVX rats [11]. On the other hand, several studies have shown the beneficial effects of coumestrol in the regulation of glucose and lipid homeostasis. In these studies, coumestrol ameliorated insulin resistance, with reduced accumulation of sphingolipids in primary rat hepatocytes [12] and downregulated adipogenic factors such as PPARγ, c/EBPα, SREBP-1, and FAS in 3T3-L1 adipocytes [13]. Interestingly, a recent study reported that coumestrol treatment reduced adiposity through the activation of brown adipose tissue (BAT) metabolism in high-fat diet (HFD)-fed male mice [14]. In this study, oral administration of coumestrol increased the mitochondrial contents of BAT, reduced visceral fat accumulation, and improved insulin sensitivity in mice. However, the metabolic effects of coumestrol in estrogen-deficient female animals and its underlying mechanisms have not yet been elucidated.

Therefore, in this study, we investigated the effects of coumestrol on adiposity, browning of white fat, and insulin signaling in HFD-fed OVX mice. Moreover, to determine the involvement of ER in the metabolic effects of coumestrol, we studied its effects when co-administered with selective antagonists of ERα and ERβ. This study provides useful information for understanding the estrogen-mimicking characteristics of coumestrol in metabolic regulation and suggests that clinical studies in postmenopausal women are warranted.

## 2. Materials and Methods

### 2.1. Animals and Surgery

Seven-week-old female C57BL/6J mice (Orient Bio Inc., Seongnam-Si, Republic of Korea) were housed under a 12 h light–dark cycle. After an acclimation period of 1 week, the mice were randomly divided into five treatment groups (7 mice per group) as follows: (1) sham control, (2) OVX control, (3) OVX + 17β-estradiol (E2), (4) OVX + coumestrol (subcutaneous administration), and (5) OVX + coumestrol (oral administration). Mice were subjected to bilateral OVX or sham operation under anesthesia with a 1.2% avertin (2,2,2-tribromoethanol) solution. Treatment with E2, coumestrol, or vehicle was initiated the day after surgery. E2 (Sigma-Aldrich, St. Louis, MO, USA) and coumestrol (ChemFaces Biochemical, Wuhan, China) were dissolved in saline containing 2% Tween-80 and 0.5% methylcellulose. Throughout the study (10 weeks), E2 was administered at 50 μg/kg by subcutaneous (SC) injection once daily. Coumestrol was administered daily at a dose of 5 mg/kg by SC injection or oral gavage. All mice were fed a phytoestrogen-free HFD (TD04059, 52% Kcal from anhydrous milk fat, Harlan Teklad, Madison, WI, USA) and water ad libitum during the experimental period. Six weeks after surgery, all OVX mice were housed singly with running wheels for 3 days, to determine voluntary running activity.

In a separate experiment using the same mouse model, coumestrol was orally administered at 10 mg/kg for 7 weeks in combination with MPP (ERα-selective antagonist) at 1 mg/kg, PHTPP (ERβ-selective antagonist) at 1 mg/kg, or ICI 182,780 (ER antagonist) at 3 mg/kg (6 mice per group). MPP, PHTPP (Tocris Bioscience, Bristol, UK), and ICI 182,780 (Sigma-Aldrich) were dissolved in corn oil and administered via SC injection once daily throughout the study. Food intake was recorded weekly, and ear temperature was measured using an infrared thermometer (IR-B153, Braintree Scientific, Braintree, MA, USA) 4 weeks after ovariectomy. At the end of the study, mice were sacrificed by overdosing with avertin. Blood was collected via cardiac puncture, and tissue samples were isolated for histological staining and molecular analysis. All animal work was carried out in accordance with institutional guidelines for the use and care of laboratory animals. The study protocol was approved by the Ethical Committee of the Andong National University (Protocol Number: 2021-1-0128-01-01).

### 2.2. Histological Staining

Sections of the parametrial adipose tissue and liver were fixed in 10% formalin, embedded in paraffin, sectioned, and stained with hematoxylin and eosin (H&E). The adipocyte area in white fat and lipid droplet area in the liver were traced and quantified using ImageJ software (Version 1.8.0, National Institutes of Health, NIH, Bethesda, MA, USA). The relative adipocyte number was calculated by dividing parametrial fat pad weight by the mean adipocyte size in each mouse (*n* = 4 per group), as previously described [15].

### 2.3. Determination of Thiobarbituric Acid Reactive Substances (TBARS)

Concentrations of TBARS in the liver were determined using a colorimetric method, as previously described [16]. Levels of TBARS were expressed as malondialdehyde (MDA) equivalents.

### 2.4. Gene Expression Analysis

Uterine tissue was homogenized in 1 mL TRIzol reagent, and total RNA was isolated. Total RNA was reverse-transcribed to cDNA using a High Capacity cDNA Reverse Transcription Kit (Applied Biosystems, Foster, CA, USA). cDNA was used as a template for the relative quantitation of selected target genes using predesigned Taq-Man primer/probe sets (Applied Biosystems). Each 20 μL reaction mixture contained 100 ng cDNA, 2 × TaqMan Fast Advanced Master Mix (Applied Biosystems), and a TaqMan primer/probe. All reactions were carried out in triplicate using a 7500 Real-Time PCR System (Applied Biosystems) under the following conditions: 95 °C for 2 min, followed by 40 cycles of 95 °C for 3 s, and 60 °C for 30 s. Results are expressed as a relative values after normalization to glyceraldehyde 3-phosphate dehydrogenase (GAPDH).

### 2.5. Western Blot Analysis

White adipose tissue (WAT), BAT, liver, and skeletal muscle tissues were homogenized in RIPA buffer containing a protease inhibitor cocktail (Sigma-Aldrich) and centrifuged to collect the supernatants. The total protein concentration was determined using the Bradford method. Equal amounts of protein were separated on a 12% sodium dodecyl sulfate-polyacrylamide gel and transferred onto polyvinylidene difluoride membranes. Membranes were blocked for 30 min in phosphate buffered saline containing 3% bovine serum albumin and 0.1% Tween-20 for 1 h at room temperature. The membranes were probed with the primary antibodies listed in Table S1, followed by incubation with the corresponding horseradish peroxidase-conjugated secondary antibodies (Sigma-Aldrich). Protein bands were visualized using enhanced chemiluminescence reagents on a Fusion Solo 6S EDGE imaging system (Vilber, Marne-la-Vallée, France) and quantified using ImageJ software (NIH).

### 2.6. Statistical Analysis

All statistical analyses were performed using one-way ANOVA using R software (4.0.4 for Windows, R Foundation, Vienna, Austria). The least squares mean option using Tukey–Kramer adjustment was used for multiple comparisons among the treatment groups. Results are expressed as mean ± SEM. *p* values < 0.05 were considered statistically significant.

## 3. Results

### 3.1. Effects of Coumestrol Treatments on Uterine Growth in OVX Mice

**Figure 1:**
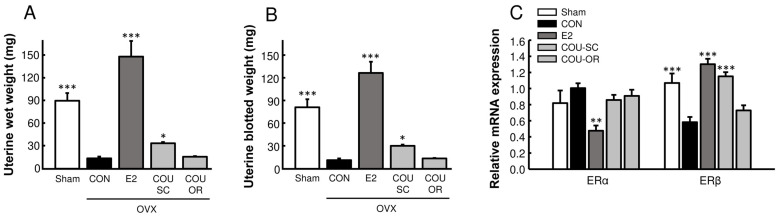
Effects of coumestrol on uterine growth in OVX mice. Mice were subjected to sham or ovariectomy surgery and received the indicated sample treatments for 10 weeks. At the end of the study, the (**A**) uterine wet and (**B**) blotted weights were recorded. (**C**) Uterine mRNA expression of estrogen receptors (ERα and ERβ). Values represent means ± SEM (*n* = 7). Con, control; E2, 17β-estradiol; COU-SC, coumestrol subcutaneous treatment; COU-OR, coumestrol oral treatment; OVX, ovariectomized. * Significantly different from the OVX control group (* *p* < 0.05, ** *p* < 0.01, *** *p* < 0.001).

As expected, ovariectomy induced uterine atrophy in mice, and E2 replacement markedly increased uterine weight in these animals (*p* < 0.001, Figure 1A,B). Coumestrol administration at 5 mg/kg for 10 weeks significantly increased uterine weight when mice were treated through the SC route but had no effect on uterine weight when mice were treated via oral gavage. Interestingly, the uterine ERα mRNA expression findings were opposite to those of uterine weight; it was slightly increased in the OVX control mice (*p* > 0.05) but significantly decreased in OVX mice exposed to E2 (*p* < 0.01, Figure 1C). Conversely, the ERβ mRNA expression findings were similar to those for uterine weight, as shown by the increased gene expression in mice exposed to E2 or SC coumestrol (*p* < 0.001).

### 3.2. Coumestrol Prevents Adiposity in HFD-Fed OVX Mice

**Figure 2:**
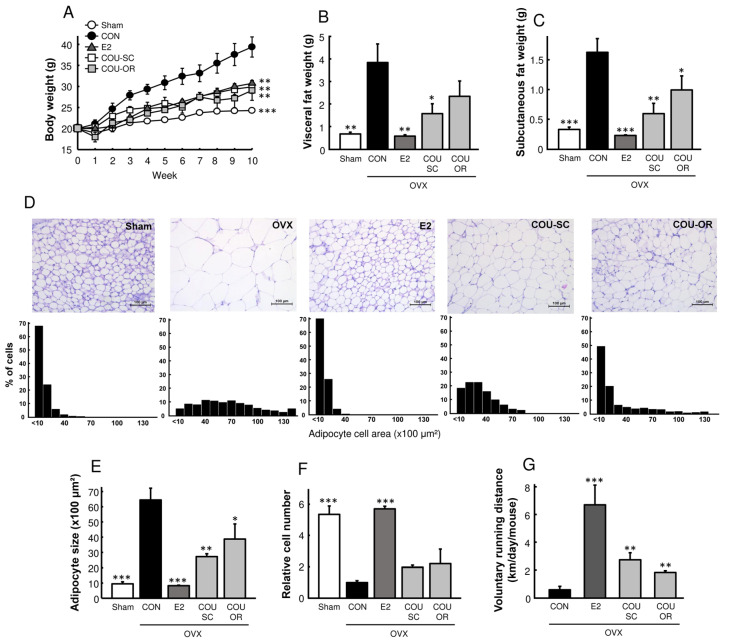
Preventive effects of coumestrol on adiposity in OVX mice fed a HFD. (**A**) Body weight and weights of (**B**) visceral and (**C**) subcutaneous adipose depots. (**D**) Representative H&E-stained section of parametrial adipose tissue and distribution histogram of adipocyte size (scale bar = 100 μm). (**E**) Average size of adipocytes and (**F**) relative adipocyte number. (**G**) At 6 weeks after surgery, all OVX mice were housed singly with running wheels, and voluntary running distance was recorded. Values represent means ± SEM (*n* = 7 for (**A**–**C**), *n* = 4 for (**D**–**G**)). CON, control; E2, 17β-estradiol; COU-SC, coumestrol subcutaneous treatment; COU-OR, coumestrol oral treatment; OVX, ovariectomized. * Significantly different from the OVX control group (* *p* < 0.05, ** *p* < 0.01, *** *p* < 0.001).

To determine the preventive effects of coumestrol on postmenopausal metabolic disorders, we used an OVX mouse model that was fed on a phytoestrogen-free HFD. Compared to the sham-operated mice, the OVX control mice exhibited a progressive increase in body weight, characterized by the accumulation of visceral and subcutaneous fat and adipocyte hypertrophy (Figure 2A–F). Coumestrol, in both SC and oral treatments, prevented adipose tissue accumulation and adipocyte hypertrophy in OVX mice. Interestingly, similarly to E2, coumestrol treatment increased the voluntary running activity in OVX mice (*p* < 0.01, Figure 2G), suggesting its anti-obesity effects were associated with enhanced physical activity.

**Figure 3:**
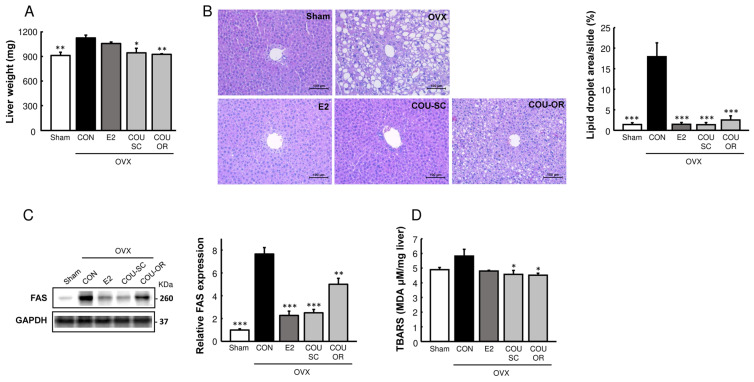
Preventive effects of coumestrol on hepatic steatosis in OVX mice fed a HFD. (**A**) Liver weight, (**B**) representative H&E-stained sections of liver (scale bar = 100 μm) with quantitative data, and (**C**) Western blot of hepatic fatty acid synthase (FAS) with quantitative data. (**D**) Concentrations of thiobarbituric acid reactive substances (TBARS) in liver were expressed as malondialdehyde (MDA) equivalents. Values represent means ± SEM (*n* = 7 for (**A**,**D**), *n* = 4 for (**B**,**C**)). CON, control; E2, 17β-estradiol; COU-SC, coumestrol subcutaneous treatment; COU-OR, coumestrol oral treatment; OVX, ovariectomized; GAPDH, glyceraldehyde-3-phosphate dehydrogenase; KDa, kilodaltons. * Significantly different from the OVX control group (* *p* < 0.05, ** *p* < 0.01, *** *p* < 0.001).

In addition, coumestrol normalized the ovariectomy-induced hepatic lipid accumulation, as shown by liver weight (*p* < 0.05) and histological analysis (*p* < 0.001, Figure 3A,B). Consistently with the hepatic lipid content, OVX control mice showed higher hepatic protein expression of fatty acid synthase (FAS), which was markedly reduced by E2 (*p* < 0.001) and coumestrol treatment (*p* < 0.01, Figure 3C). The hepatic level of TBARS, a marker of oxidative stress, was increased by ovariectomy, but normalized by coumestrol treatment (Figure 3D). These results demonstrate that coumestrol prevents adiposity and improves hepatic lipid homeostasis in HFD-fed OVX mice.

### 3.3. Coumestrol Modulates Protein Expressions Involved in Browning of White Fat

**Figure 4:**
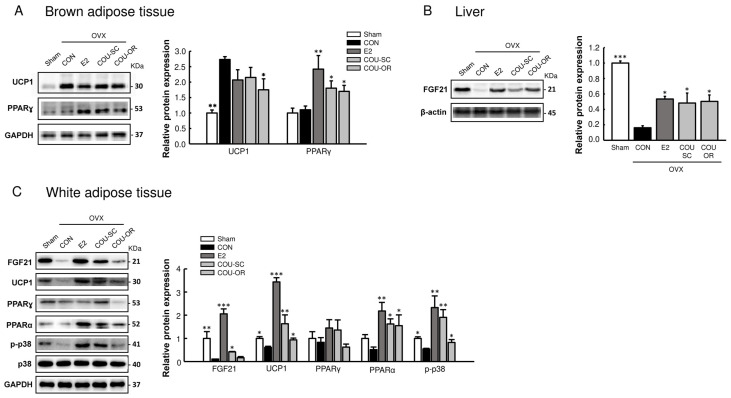
Regulatory effects of coumestrol on the browning of white fat in OVX mice. Western blotting of (**A**) uncoupling protein 1 (UCP1) and peroxisome proliferator-activated receptor-γ (PPARγ) in brown adipose tissue; (**B**) fibroblast growth factor 21 (FGF21) in the liver; and (**C**) FGF21, UCP1, PPARγ, PPARα, phosphorylated p38 (p–p38), and total p38 in white adipose tissue with quantitative data. Values represent means ± SEM (*n* = 4). CON, control; E2, 17β-estradiol; COU-SC, coumestrol subcutaneous treatment; COU-OR, coumestrol oral treatment; OVX, ovariectomized; GAPDH, glyceraldehyde-3-phosphate dehydrogenase; KDa, kilodaltons. * Significantly different from the OVX control group (* *p* < 0.05, ** *p* < 0.01, *** *p* < 0.001).

Browning of white fat has been recognized as a key strategy to restrict obesity and obesity-related disorders [17]. It has been demonstrated that a peroxisome proliferator-activated receptor (PPAR) α-driven increase in fibroblast growth factor 21 (FGF21) plays a central role in the conversion of white into brown-like adipocytes [18,19]. In this study, similarly to E2, coumestrol increased FGF21 protein expression in the liver and the WAT of OVX mice (*p* < 0.05, Figure 4). In WAT, uncoupling protein 1 (UCP1), PPARα expression, and p38 phosphorylation were reduced following ovariectomy, but increased after E2 and coumestrol treatments (Figure 4C). In BAT, PPARγ protein expression was increased by E2 (*p* < 0.01) and coumestrol (*p* < 0.05) treatments, whereas UCP1 expression was slightly decreased (*p* < 0.05 only for coumestrol oral treatment, Figure 4A).

### 3.4. Coumestrol Normalizes the Phosphorylation of PI3K and Akt in the Skeletal Muscle and Liver of OVX Mice

**Figure 5:**
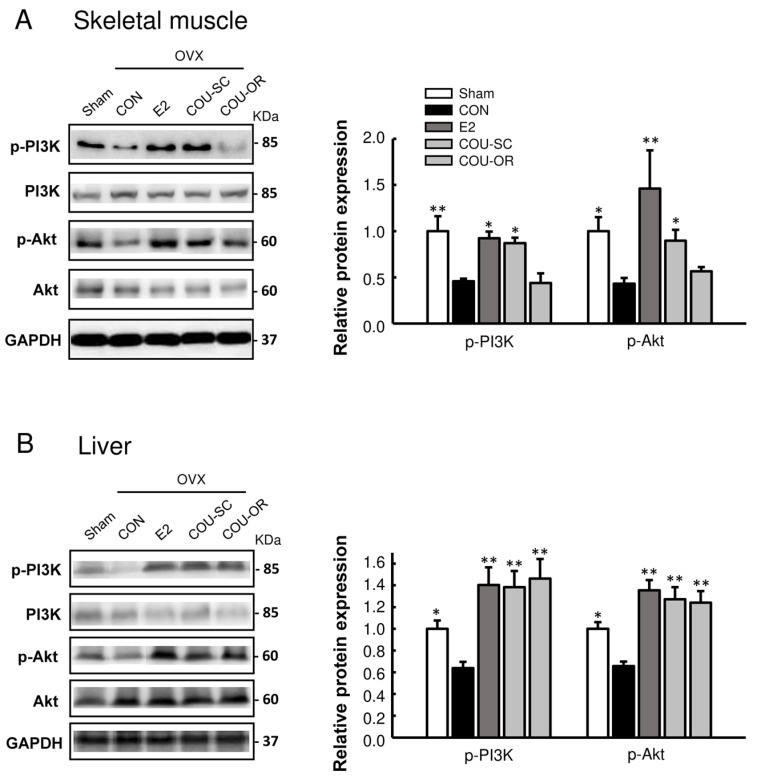
Regulatory effects of coumestrol on insulin signaling in OVX mice. Western blotting of (**A**) phosphorylated phosphoinositide 3-kinase (p-PI3K), total PI3K, phosphorylated protein kinase B (p-Akt), and total Akt in skeletal muscle and (**B**) liver with quantitative data. Values represent means ± SEM (*n* = 4). CON, control; E2, 17β-estradiol; COU-SC, coumestrol subcutaneous treatment; COU-OR, coumestrol oral treatment; OVX, ovariectomized; GAPDH, glyceraldehyde-3-phosphate dehydrogenase; KDa, kilodaltons. *Significantly different from the OVX control group (* *p* < 0.05, ** *p* < 0.01).

The phosphoinositide 3-kinase (PI3K)/protein kinase B (Akt) pathway is a key regulator of insulin signaling, resulting in an increased glucose uptake in skeletal muscles and reduced glucose production in the liver [20]. Our study showed that ovariectomy reduced the phosphorylation of PI3K and Akt in skeletal muscle and liver, which was normalized by E2 treatment (Figure 5). Phosphorylation of these proteins was normalized in the skeletal muscle using SC treatment with coumestrol (*p* < 0.05), and in the liver with both SC and oral treatments (*p* < 0.01), suggesting an improvement of glucose homeostasis from coumestrol in OVX mice.

### 3.5. Metabolic Effects of Coumestrol in OVX Mice Are Primarily Mediated by ERβ

**Figure 6:**
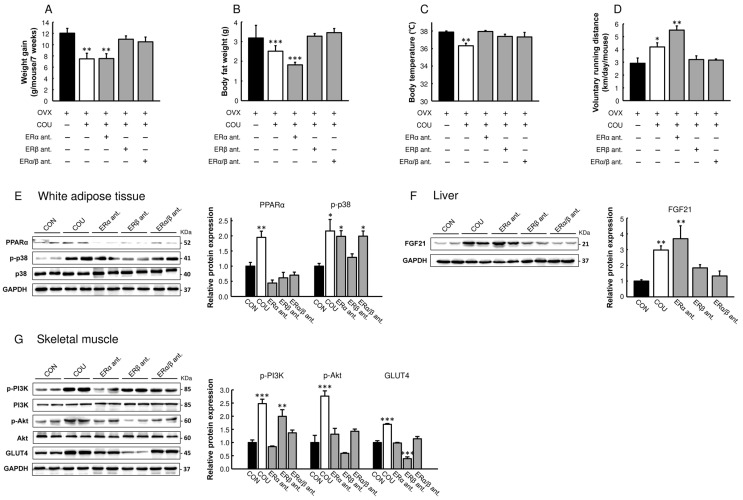
ER-mediated actions of coumestrol in the regulation of energy homeostasis. OVX mice were orally administered with 10 mg/kg coumestrol in combination with MPP (ERα-selective antagonist), PHTPP (ERβ-selective antagonist), or ICI 182,780 (ER antagonist) for 7 weeks. (**A**) Weight gain and (**B**) body fat weight. (**C**) Body temperature was measured in the ear using an infrared thermometer 4 weeks after ovariectomy. (**D**) Voluntary running distance was recorded 6 weeks after surgery. Protein expression of (**E**) PPARα, p-p38, and p38 in white adipose tissue, (**F**) FGF21 in the liver, and (**G**) p-PI3K, PI3K, p-Akt, Akt, and GLUT4 in skeletal muscle were measured by Western blotting. Values represent means ± SEM (*n* = 6 for A-D, *n* = 4 for E-G). CON, control; COU, coumestrol; ERα ant., ERα-selective antagonist; ERβ ant., ERβ-selective antagonist; ERα/β ant., ERα/β antagonist; OVX, ovariectomized; GAPDH, glyceraldehyde-3-phosphate dehydrogenase; KDa, kilodaltons. * Significantly different from the OVX control group (* *p* < 0.05, ** *p* < 0.01, *** *p* < 0.001).

As coumestrol showed estrogen-mimetic effects on metabolic parameters in OVX mice, we examined whether the effects of coumestrol were dependent on the ER (Figure 6). In a separate animal study, OVX mice received coumestrol orally in combination with an SC injection of an ERα-selective antagonist, ERβ-selective antagonist, or ER antagonist. As expected from the previous experiments, coumestrol protected OVX mice against increased body weight, fat accumulation, elevated body temperature, and reduced voluntary activity (Figure 6A–D). In contrast, the protective effects of coumestrol were lost when mice were treated with an ERβ antagonist. In addition, the increased protein expression of p-p38 in WAT and FGF21 in the liver induced by coumestrol was reversed by the ERβ antagonist (Figure 6E,F). The protein expression of PPARα in WAT, and that of p-AKT and GLUT4 in skeletal muscle, was increased by coumestrol but reversed by both ERα and ERβ antagonists (Figure 6E,G). These findings indicate that coumestrol improves lipid and glucose homeostasis via ERβ in OVX female mice, although ERα is also involved, according to the target tissues.

## 4. Discussion

We recently studied the estrogenic activities of 15 known phytoestrogens, using an in vitro ER transactivation assay and an in vivo uterotrophic assay, and found that coumestrol and 8-prenylnaringenin had the most potent estrogenic properties [6]. Therefore, we evaluated the protective effects of coumestrol on menopausal metabolic disorders and its mechanism of action in estrogen-deficient female mice. The current results showed that coumestrol could prevent adiposity and improve the signal transduction involved in the browning of white fat and insulin sensitivity in HFD-fed OVX mice. These beneficial effects of coumestrol were comparably observed when mice were treated through the SC and oral routes. The improved insulin signal transduction in muscle and liver with coumestrol observed in this study is consistent with a previous report, wherein coumestrol improved glucose tolerance and increased hepatic glycogen content in type-2 diabetic mice [21]. In addition, Kim et al. [14] recently reported that coumestrol exerted anti-obesity effects through the activation of BAT metabolism in male mice. Thus, these studies support the current finding that coumestrol improves energy metabolism in OVX female mice. Notably, the protein expression of FGF21, which is a key regulator in maintaining whole-body energy balance and protecting the liver from steatosis [22], was markedly decreased in the liver and WAT following ovariectomy, but was restored by E2 and coumestrol treatments (Figure 4). Serum FGF21 is primarily secreted by the liver and exerts its endocrine action on the central nervous system and adipose tissue [22]. It has been demonstrated that E2 replacement in OVX mice increases hepatic FGF21 production and enhances energy expenditure [23,24], suggesting that FGF21 is an important mediator in the regulation of energy homeostasis by E2. Therefore, our results suggest that coumestrol exerts estrogen-mimetic effects in the control of energy homeostasis, in part by stimulating hepatic FGF21 production. Moreover, because FGF21 is known to modulate the hepatic metabolic pathway to suppress steatosis [25], the observed reduced FAS expression and lipid accumulation by E2 and coumestrol in the liver (Figure 3) might be associated with increased FGF21 production.

It has been suggested that coumestrol may have favorable effects on insulin sensitivity. In insulin-resistant hepatocyte, coumestrol suppressed sphingolipid accumulation through inhibition of the ceramide de novo synthesis pathway [12]. In cultured muscle cells, coumestrol promoted mitochondrial biogenesis and ATP synthesis in a Sirt1-dependent manner [26]. Since mitochondrial dysfunction is known to be closely related with the pathogenesis of insulin resistance, this result suggests a novel mechanism of coumestrol in the muscular regulation of insulin sensitivity. In addition, dietary treatment of coumestrol decreased the levels of fasting blood glucose and HbA1c and improved glucose tolerance in a genetically-induced mouse model of diabetes, although the underlying mechanism was not studied [21]. Alongside this evidence, we here observed that coumestrol increased the phosphorylation of PI3K and Akt—the key regulators of the insulin signaling pathway—in the skeletal muscle and liver of OVX female mice (Figure 5). It was demonstrated that a damaged PI3K/Akt pathway in various tissues, such as skeletal muscle, liver, adipose tissue, pancreas, and brain, leads to insulin resistance [20]. Thus, these findings support the beneficial effects of coumestrol as a modulator of insulin sensitivity and glucose homeostasis. However, further studies are needed to elucidate more clearly its mechanism of action.

It has been shown that ERα and ERβ have distinct actions in the regulation of energy homeostasis and that their action is tissue-specific in the brain, skeletal muscle, adipose tissue, liver, and pancreas [27,28]. The interaction of coumestrol with the ER has been studied using in vitro, in vivo, and in silico methods. The majority of these studies found that, similarly to other phytoestrogens, coumestrol has a greater selective affinity for ERβ than ERα, although its affinity is weaker than that of E2 [2,29,30,31]. Consistently, our results demonstrated that the metabolic effects of coumestrol in OVX mice were abolished with an ERβ-selective antagonist but not with an ERα-selective antagonist, although the protein expression data showed that ERα is also involved in the mechanism of action of coumestrol (Figure 6). These findings indicate that the metabolic action of coumestrol is ERβ-dependent. In addition, Fekri et al. [32] recently reported that the memory enhancing and anxiolytic effects of coumestrol were reversed by treatment with an ERβ-selective antagonist in a male mouse model of chronic restraint stress. In their study, the antioxidant and anti-apoptotic effects of coumestrol in brain tissue were lost following co-administration with an ERβ antagonist. Therefore, alongside our current data, these results suggest that ERβ-mediated actions of coumestrol occur in metabolic tissues, including the brain, in both male and female rodents. However, it is also important that coumestrol, in addition to classical ER signaling, may act via other cellular pathways. In OVX rats, the neuroprotective effects of E2 were abolished by ICI 182,780 (ERα/ERβ antagonist) administration, whereas the effects of coumestrol were only partially abolished by the antagonist [8]. Thus, additional research using an ER knockout animal model is needed, to determine the ER-dependent and ER-independent mechanisms of action of coumestrol.

As the uterine response to estrogens involves the activation of a large spectrum of estrogen-responsive genes, the uterus has been used as a classical target organ to determine the estrogenicity of natural and synthetic compounds [33]. In this study, coumestrol treatment with 5 mg/kg SC injection significantly increased uterine weight in OVX mice, but oral administration did not (Figure 1). Consistent with these changes in uterine growth, ERβ gene expression in the uterus was increased by E2 and SC coumestrol treatments. Although we failed to observe an uterotrophic effect of coumestrol following oral administration, previous short-term studies using a higher oral dose (50–100 mg/kg) of coumestrol showed an obvious uterotrophic effect in immature intact, immature OVX, and mature OVX rats [6,34]. Moreover, in our pilot study, oral administration of coumestrol at 10 mg/kg significantly increased uterine weight in OVX mice. Furthermore, Markaverich et al. [35] found that acute (SC injection) or chronic (oral administration) treatment with coumestrol significantly increased uterine weight but failed to cause cytosolic ER depletion and nuclear ER accumulation in immature OVX rats, suggesting a lack of true estrogenic activity from coumestrol. However, in our previous study, the uterotrophic effect of coumestrol was completely abolished by co-treatment with an ER antagonist, and coumestrol showed strong ER-mediated transcriptional activity in a luciferase reporter gene assay using VM7Luc4E2 cells (a variant of MCF-7 human breast cancer cells) [6]. Taken together, coumestrol is believed to act as an ER agonist, although its activity can vary depending on the target tissue and experimental conditions (administration dosage, route, etc.).

Through identifying the biological activities of coumestrol, several studies have focused on increasing the coumestrol content in its plant sources. Recently, Ohta et al. [36] investigated the optimal cultivation conditions for producing soybean sprouts with high coumestrol contents. In their study, they found that the content of coumestrol depended on temperature but not on light, bacteria, and sprout color. In addition, Fields et al. [37] reported that alfalfa plants inoculated with stemphylium had a higher coumestrol content than uninfected samples; however, there was no change in coumestrol in response to aphids or development stage. In general, the use of phytoestrogens, including coumestrol, has been limited, due to their low concentrations in plant sources. Therefore, these studies will help to extend their industrial application for use as a therapeutic agent.

In conclusion, the current study demonstrated that coumestrol exhibits estrogen-mimetic effects in preventing metabolic dysfunctions such as adiposity and defects in cellular signaling involved in white fat browning and muscle insulin sensitivity in OVX mice. Importantly, the metabolic regulation of coumestrol was primarily mediated by ERβ. These findings provide novel insights into the mechanisms underlying the beneficial effects of coumestrol in postmenopausal treatment and warrant further studies to validate its effects in a broad spectrum of postmenopausal diseases.

## Data Availability

The datasets used and/or analyzed during the current study are available from the corresponding author upon reasonable request.

## Supplementary Materials

The following supporting information can be downloaded at: https://www.mdpi.com/article/10.3390/nu15040954/s1, Table S1: Antibodies used for western blotting.
