# Supplementary material for: Protective Effects of Coumestrol on Metabolic Dysfunction and Its Estrogen Receptor-Mediated Action in Ovariectomized Mice
Source: Nutrients. 2023 Feb 14;15(4):954. doi: 10.3390/nu15040954 (PMC9966481; doi:10.3390/nu15040954)
Supplement: Supplementary file 1 [file nutrients-15-00954-s001.zip › Table S1.pdf]

Table S1. Antibodies used for western blotting.

Primary antibody	Clone	Company	Catalog No.	Dilution
**FGF21**	Monoclonal	Thermo Fisher	MA5-32652	1:1000
**UCP-1**	Monoclonal	Cell Signaling	#14670	1:1000
**PPARα**	Monoclonal	ABcam	a126285	1:2000
**PPARɣ**	Monoclonal	ABcam	ab178860	1:2000
**p38**	Polyclonal	Cell Signaling	#9212	1:1000
**p-p38**	Polyclonal	ABcam	ab47363	1:2000
**PI3K**	Monoclonal	Thermo Fisher	MA1-74183	1:1000
**p-PI3K**	Polyclonal	Thermo Fisher	PA5-104853	1:1000
**AKT**	Polyclonal	Cell Signaling	#9272	1:1000
**p-AKT**	Polyclonal	Cell Signaling	#9271	1:1000
**FASN**	Polyclonal	Thermo Fisher	PA1-32355	1:1000
**GLUT4**	Monoclonal	ABcam	ab188317	1:1000
**FGF21**	Monoclonal	Thermo Fisher	MA5-32652	1:1000
**GAPDH**	Monoclonal	Abbkine	A01020-SK	1:2000
**β-actin**	Monoclonal	Abbkine	A01010-SK	1:2000
